# Geospatial Analysis of Dental Access and Workforce Distribution in Kenya

**DOI:** 10.5334/aogh.3903

**Published:** 2022-11-21

**Authors:** Brenda A. Okumu, Marc Tennant, Estie Kruger, Arthur M. Kemoli, Frank A. Roberts, Ana L. Seminario

**Affiliations:** 1 Department of Community and Preventive Dentistry and Periodontology, School of Dentistry, Moi University, Eldoret, KE; 2 Faculty of Science, School of Human Sciences, Co-director International Research Collaborative – Oral Health and Equity (IRCOHE). University of Western Australia, AU; 3 Dental Public Health, Faculty of Science, School of Human Sciences, Co-director International Research Collaborative – Oral Health and Equity (IRCOHE). University of Western Australia, AU; 4 Department of Paediatric Dentistry & Orthodontics, University of Nairobi. Member of the Board, Timothy A. DeRouen Center for Global Oral Health, University of Washington, Seattle, US; 5 Regional Initiatives in Dental Education (RIDE) Program, Associate Dean for Regional Affairs, University of Washington, US; 6 Global Health, School of Public Health, University of Washington, US; 7 School of Dentistry, Universidad Peruana Cayetano Heredia, Lima, Peru

**Keywords:** dental workforce distribution, dentists, dental access, urbanization, population size, Geographical Information System, Kenya

## Abstract

**Background and Objective:**

One of the major factors affecting access to quality oral healthcare in low- and middle-income countries is the under-supply of the dental workforce. The aim of this study was to use Geographical Information System (GIS) to analyse the distribution and accessibility of the dental workforce and facilities across the Kenyan counties.

**Methods:**

This was a cross-sectional study targeting dental professionals and their practices in Kenya in 2013. Using QGIS 3.16, these data were overlaid with data on population size and urbanization levels. For access measurement, buffers were drawn around each clinic at distances of 2.5, 5, 10 and 20 km, and the population within each determined.

**Findings:**

Nine hundred six dental professionals in 337 dental clinic locations were included in the study. Dentists, community oral health officers (equivalent to dental therapists) and dental technologists comprised 72%, 15% and 12%, respectively. Nairobi county with 100% urbanization and >4000 people/km^2^ had 43% of the workforce and a dentist to population ratio of 1:9,018. Wajir with an urbanization level of 15% and 12 people/km^2^ had no dental facility. Overall, 11%, 19%, 35% and 58% of the Kenyan population were within 2.5, 5, 10 and 20 km radius of a dental clinic respectively.

**Conclusion:**

Maldistribution of dental workforce in Kenya persists, particularly in less urbanized and sparsely populated areas. GIS map production give health planners a better visual picture of areas that are most in need of health care services based on population profiles.

## Background

Oral diseases are a major global public health issue, with the associated global burden of the diseases (GBDs) estimated at 3.5 billion people, mostly due to dental caries and periodontal disease [1,2]. What is even more worrying is the widening inequalities in the prevalence of these oral diseases, with low- and middle-income countries (LMICs) having the highest burden of untreated disease compared to high-income countries (HICs) [1]. This disparity is largely dependent on the performance of healthcare systems, and based on how accessible, equitable, effective, relevant, and socially acceptable the health services are to the target population [3]. The high performance seen in many HICs has been attributed to better financing systems, parity in service delivery, adoption of promotive and preventive health, and better monitoring and healthcare regulation structures [4]. At the population level, higher education attainment in these countries has been associated with better health outcomes [5].

One of the major factors affecting access to quality oral healthcare in LMICs is the supply and distribution of professionals who are qualified to offer dental health services – the oral health workforce (OHW). Other than a few jurisdictions in Europe, the US and Australia that have reported adequate or an oversupply of dentists due to increased training and registration [6,7], the shortage of dentists and other allied professionals appears to be global [8]. The World Health Organization (WHO) estimates a global shortage of 4.3 million health workers, with the countries in sub-Saharan Africa being the most affected [9]. Kenya, a low-middle income country (LMIC), is one of the 57 countries listed by the WHO as having critical shortage of health care workers. Historically, the demand for oral health care in the East African Country has always surpassed the supply of its OHW who include dentists, community oral health officers (COHOs) and dental technologists (DTs). Dentists are the primary oral health care providers and constitute the majority of the OHW. At the time of this study, the dentist to population ratio was at 1:42,000, significantly below the WHO recommended ratio of 1:7,000 [10,15]. COHOs (Dental Therapists) on the other hand are diploma holders who have undergone a three-year prescribed clinical and dental public health training. Their scope of practice includes providing community dental public information, limited clinical care that include restorations especially in primary teeth, application of fluoride, sealants especially using GIC, temporary or intermediate restorations, oral prophylaxis and oral health education. They are often placed in primary health care facilities, where they are also expected to lead oral health demonstrations, exhibitions and education to communities and schools [11]. However, majority still end up working with dentists at higher levels of care located in major cities and towns. DTs are also diploma holders and are trained to design, fabricate and fit dental restorations, prostheses and appliances respectively. This implies that they work in settings where dentists are likely to practice. Mandated to regulate dental practice and ensure compliance to high standards of care is the Kenya Medical Practitioners and Dentists Council (KMPDC). The body ensures that all practioners and health facilities are duly registered and licensed and have their scope of work defined (https://kmpdc.go.ke/vision-missionmandate/).

Shortages of OHW implies that there is limited availability to quality care and existence of inequalities between rural and urban areas [12,13]. The National Oral Health Survey Report of 2015 for Kenya, cited these as the major risk factors in the high prevalence of dental disease, with 34% and 98% of the adult population surveyed having untreated dental caries and gum disease, respectively [10]. Compared to the majority of urban dwellers, most rural dwellers in Kenya have been found to be financially poorer [14], having limited access to education, employment opportunities, communication networks and health facilities. This makes the rural dwellers struggle with out-of-pocket payments for oral health services as they also lack health insurance coverage and have to cover longer distances to access the limited facilities and oral healthcare providers [5,8].

In an attempt to address the perennial inequities in health care that were associated with a centralised system of government, Kenya in 2013 devolved its health care functions, including management of human resource for health (HRH) and public health facilities, from the National to the newly formed 47 county governments. However, several reports have shown that most counties are still faced with human resource deficiencies and inadequate funding, among other challenges [16,17]. Counties with larger rural populations are unable to attract specialist workforce such as dentists due to poor infrastructure and limited opportunities for career growth. Further, oral health in Kenya continues to be under prioritized in budgetary allocations. For instance, in 2013 the national government allocated less than 1% of the 4.5% total health expenditure to oral healthcare [10]. Although oral health care is recognized as one of the health aspect to be included within the Universal Health Care (UHC), in the current state, it is not mentioned as a key area [18]. It remains to be seen whether these efforts will significantly boost health care access and in particular oral health care.

In this study, we used Geographical Information System (GIS) technology to analyse the distribution of the dental workforce and the dental facility locations in Kenya before the devolution of health care system. The technology is well suited for evaluating and understanding geographical access to health services and has the capacity to show many different kinds of data on one map. As a result, it gives health planners a geographical picture of the need and type of healthcare services based on population profiles [19]. Previously, Kenya has only relied on the number of registered dentists to calculate Dentist/general population ratio without taking into consideration the national distribution of its population, hence not reliable for any future projections and effective workforce planning [20,21]. We believe that health authorities at both the national and the newly formed county governments will find the visual information helpful to better understand the shortages and imbalances of OHW and dental facilities for appropriate decisions in workforce planning.

## Methods

### Study design and population

This was a descriptive cross-sectional study targeting all dental professionals (dentists, COHOs [dental therapists], and dental technologists [DTs]) practicing in Kenya in 2013, the year when Kenya transitioned into a two-tier devolved system of government. All dental professionals registered by the Ministry of Health (MOH) in 2013 and all dentists who were on the 2013 retention registers of the KMPDC were included in the study. The MOH data was retrieved from the ministry headquarters, oral health section and we verified that this was the actual data used in the identification of the number and location of dental professionals in public service, and for workforce planning. We further counter-checked the MOH data with the KMPDC register to verify the accuracy of the entries.

Data on the Kenyan population was derived from the 2009 Kenya population and housing census, the census closest to our dental professional data that was available on the Kenya Bureau of Statistics website (https://www.knbs.or.ke/publications/). Shape files (formats that store vector data including location, shape, and attributes of geographic features) and global positioning data (longitudes and latitudes) of the practice locations were retrieved from open access websites, including https://africaopendata.org/dataset/kenya-counties-shapefile.

### Data management and analysis

The geospatial analysis was done using Quantum Geographic Information System (QGIS) version 3.16.9. The data for dental professionals and dental clinic locations were first recorded in spreadsheet software (Excel 2016, Microsoft Corp., Redmond, WA, USA). Geocoding of the physical practice addresses of all the dental professionals whose locations could be determined was completed using Google Maps. The coordinates were then mapped nationwide across all the counties and overlaid with census data on population size and urbanization levels of counties. The distribution of dentists, COHOs and DTs, and their practice location was then analyzed based on these three variables per county.

For access measurements, geographic tools in QGIS were used to draw buffers around each of the dental clinics/facilities at specific distances. First, a random distribution of pink dots (Figure 4), each representing 500 people was added to the map based on the total population of each ‘boundary region’ at the location level. Concentric buffers were then made around each clinic (Figure 4, yellow dots) that had at least one dental professional at increasing distances of 2.5, 5, 10 and 20 km. Subsequently, a spatial query was completed to determine which populations were within each of these distances (number of dots multiplied by 500) and this data was saved in Excel as a CSV file for further analysis. The percentage population within each of the distances was then analyzed per county.

Ethics approval was not deemed necessary because only secondary data with no identifying information was used.

## Results

A total of 906 dental professionals in 337 public and private dental facility/clinic locations were included in the study. The majority of oral health providers were dentists (n = 658, 72%) followed by COHOs (n = 137, 15%) and DTs (n = 111, 12%). At the national level, Kenya had a population density of 66 people/km^2^, with a dentist- population ratio of approximately 1:50,000 and urbanization of 32%. Supporting Table 1 gives a summary of all the county data analyzed in this study.

**Supporting Table 1:** Summary of Counties by Variables Analysed.


COUNTY	DENTISTS	COHOS	DTS	OHW (ALL)	DENTAL FACILITIES	POP. DENSITY (PER KM^2^)	DENTIST: POP. RATIO	URBANISATION (%)

BARINGO	3	6	1	10	4	50.44	185187	11

BOMET	3	3	1	7	4	293.04	243376	15

BUNGOMA	5	4	3	12	6	453.49	275013	22

BUSIA	2	3	1	6	4	438.9	371973	16

ELGEYO	2	1	1	4	1	122.12	184999	14

EMBU	7	1	1	9	3	183.18	73745	16

GARISSA	3	0	1	4	1	14.1	207687	24

HOMA BAY	2	2	1	5	2	302.77	481897	14

ISIOLO	1	1	0	2	1	5.66	143294	44

KAJIADO	6	2	2	10	4	31.38	114552	41

KAKAMEGA	7	1	3	11	2	544.26	237236	15

KERICHO	7	3	2	12	4	305.91	107485	39

KIAMBU	29	6	6	41	17	638.23	55975	61

KILIFI	8	2	3	13	5	88.01	138717	26

KIRINYAGA	5	2	2	9	3	357.01	105611	16

KISII	5	2	1	8	3	874.58	230456	22

KISUMU	17	2	3	22	8	464.5	56995	52

KITUI	3	4	2	9	3	33.21	337570	14

KWALE	2	2	1	5	2	78.59	324966	18

LAIKIPIA	4	1	1	6	3	42.19	99807	25

LAMU	1	1	0	2	1	16.19	101539	20

MACHAKOS	14	5	4	23	4	176.96	78470	52

MAKUENI	4	4	1	9	4	110.45	221132	12

MANDERA	0	1	0	1	1	39.47	0	18

MARSABIT	1	2	0	3	2	4.1	291166	22

MERU	7	6	2	15	6	195.54	193757	12

MIGORI	1	2	1	4	1	353.24	917170	34

MOMBASA	41	4	4	49	24	4292.09	22912	100

MURANGA	5	3	1	9	3	368.37	188516	16

NAIROBI	348	18	25	391	148	4514.96	9018	100

NAKURU	23	7	7	37	12	213.92	69710	46

NANDI	4	1	0	5	2	261.07	188241	14

NAROK	1	1	1	3	1	47.45	850920	7

NYAMIRA	1	0	0	1	1	665.22	598252	14

NYANDARUA	4	2	3	9	1	183.74	149067	19

NYERI	22	3	4	29	13	207.83	31525	25

SAMBURU	1	0	0	1	1	10.65	223947	17

SIAYA	3	3	1	7	4	332.88	280768	11

TAITA	3	3	2	8	3	16.66	94886	23

TANA RIVER	1	1	0	2	2	6.25	240075	15

THARAKA	3	2	0	5	3	138.44	121777	7

TRANS NZOIA	3	1	2	6	2	328.09	272919	20

TURKANA	0	1	1	2	1	12.45	0	14

UASIN GISHU	42	16	15	73	13	267.3	21290	39

VIHIGA	2	1	0	3	1	1044.68	277311	31

WAJIR	0	0	0	0	0	11.68	0	15

WEST POKOT	2	1	1	4	2	55.91	256345	8

**KENYA**	**658**	**137**	**111**	**906**	**337**	**66**	**52388**	**32.3**


### Distribution of oral health workers (OHWs) in the counties

**Figure 1:**
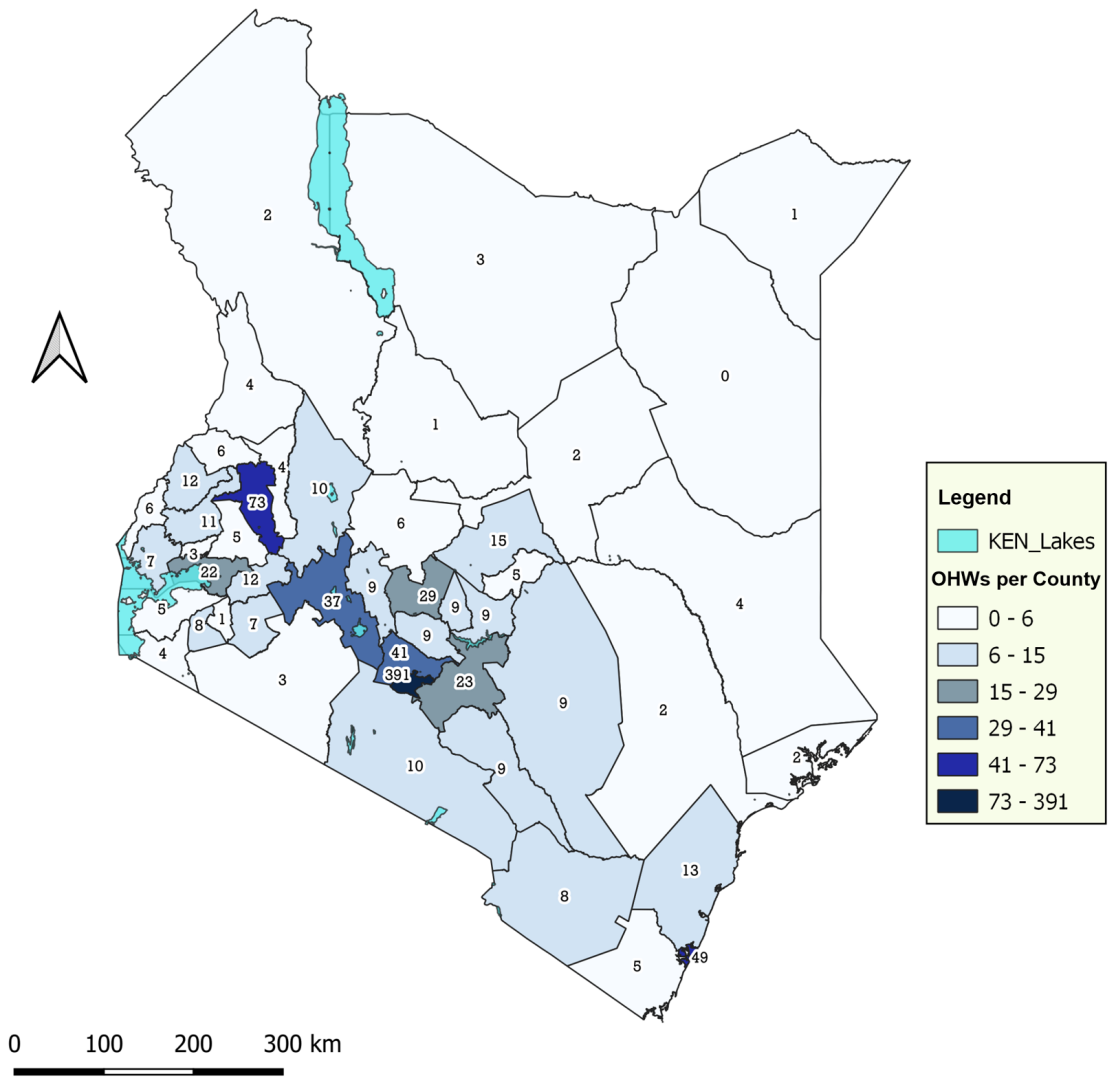
The number of oral health workers per county.

The highest population of dental workers was within counties in the middle third of the country and to a lesser extent, the coastal region. Nairobi City County recorded the highest number of OHWs (n = 391, 43%), followed by Uasin Gishu at a distant second (n = 73, 8%). Mombasa came in third with only 5% of the OHWs (n = 49). On the other hand, the least number of OHWs was in counties in the northern and north-eastern regions, including Wajir County (n = 0), Mandera County (n = 1), and Samburu County (n = 1). Generally, more than 70% of the 34 counties had 10 or fewer dental professionals (Figure 1).

### Distribution of dentists by population size and density

**Figure 2:**
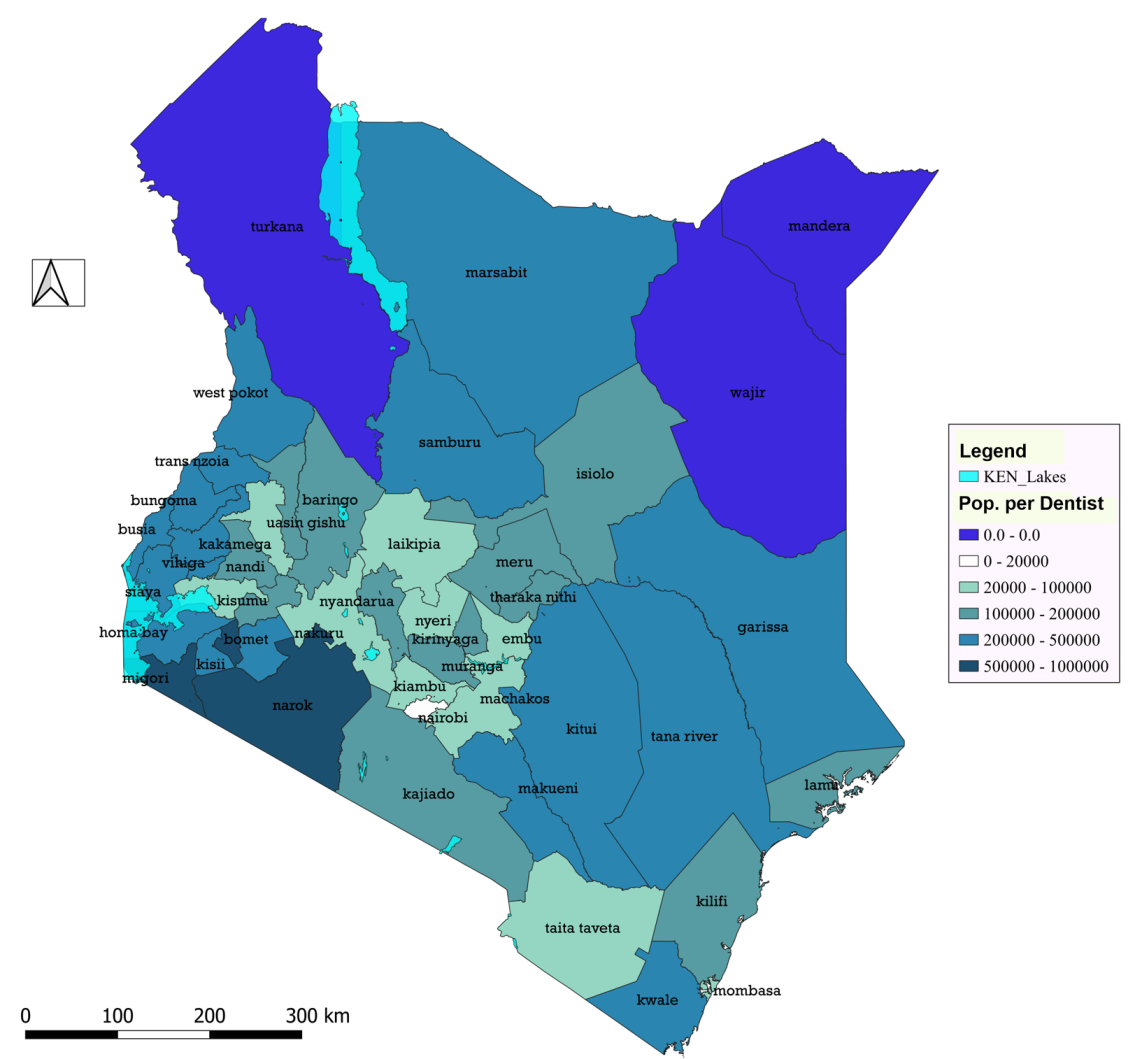
The dentist to population ratio in Kenyan counties.

Counties situated in the middle third of the country recorded the highest dentist to population ratios (Figure 2). Nairobi and Mombasa counties, with a population density of > 4000 people/km^2^, had the highest ratios of 1:9,000 and 1:22,000, respectively. Conversely, counties situated to the north and parts of the southern regions had some of the lowest dentist to population ratios. For instance, Marsabit County with a population density of 4 people/km^2^ had a ratio of 1:290,000 and Narok County with a population density of 47 people/km^2^ had the lowest ratio of 1:850,000. Furthermore, three counties were without a single dentist: Turkana (13 people/km^2^), Wajir (12 people/km^2^), and Mandera (39 people/km^2^). On the other hand, Uasin Gishu and Nyeri counties, both with a density of less than 300 people/km^2^ had relatively high ratios of 1:21,000 and 1:31,000, respectively.

### Distribution of dental clinics by county urbanisation levels

**Figure 3:**
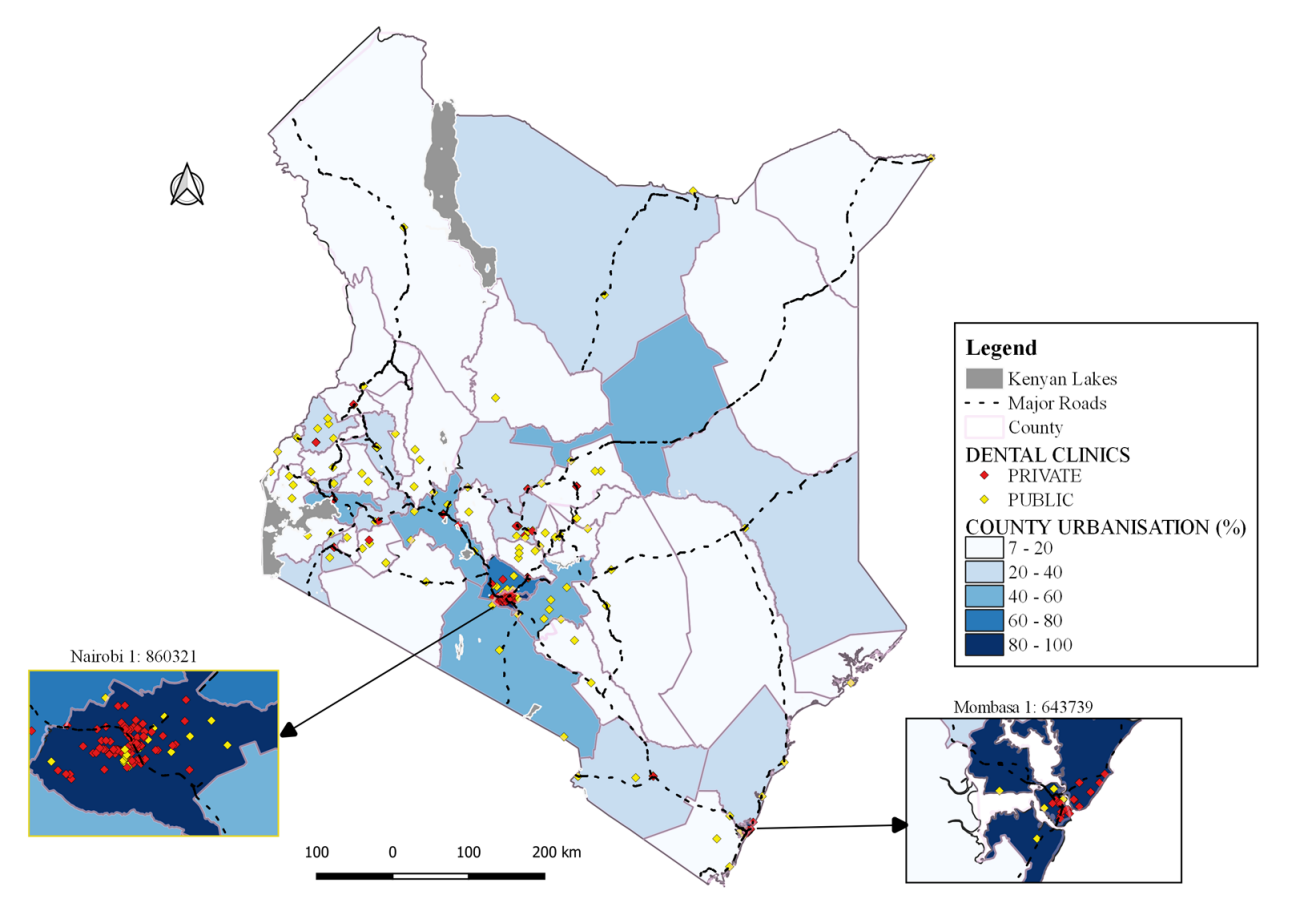
Distribution of dental clinics by county urbanization levels.

Counties in the middle third of the country and to a lesser extent the coastal region had the highest number of dental facilities and were more urbanized (Figure 3). Nairobi City and Mombasa (Figure 3, inset), being 100% urbanized, had the highest number of dental clinics (n = 148 and 24, respectively), and Kiambu with the second highest rate of 61% had among the highest number of dental facilities (n = 17). Conversely, counties within the lowest urbanization quartile (<20%) had the lowest number of dental clinics. Wajir at 15% urbanization did not have a dental facility, whereas Narok and Tharaka Nithi counties, which had the lowest urbanization rates (7%) had only 1 and 3 facilities, respectively. Uasin Gishu county with an urbanization level of 39% had among the highest numbers of facilities (n = 13).

Within the highly urbanized counties, the number of private clinics (Figure 3, red stars) significantly outnumbered the public facilities (Figure 3, yellow stars). For instance, Nairobi at 100% urbanization had more private clinics (n = 124, 84%) compared to public facilities (n = 24, 16%).

### Measure of access to dental services by distance

**Figure 4:**
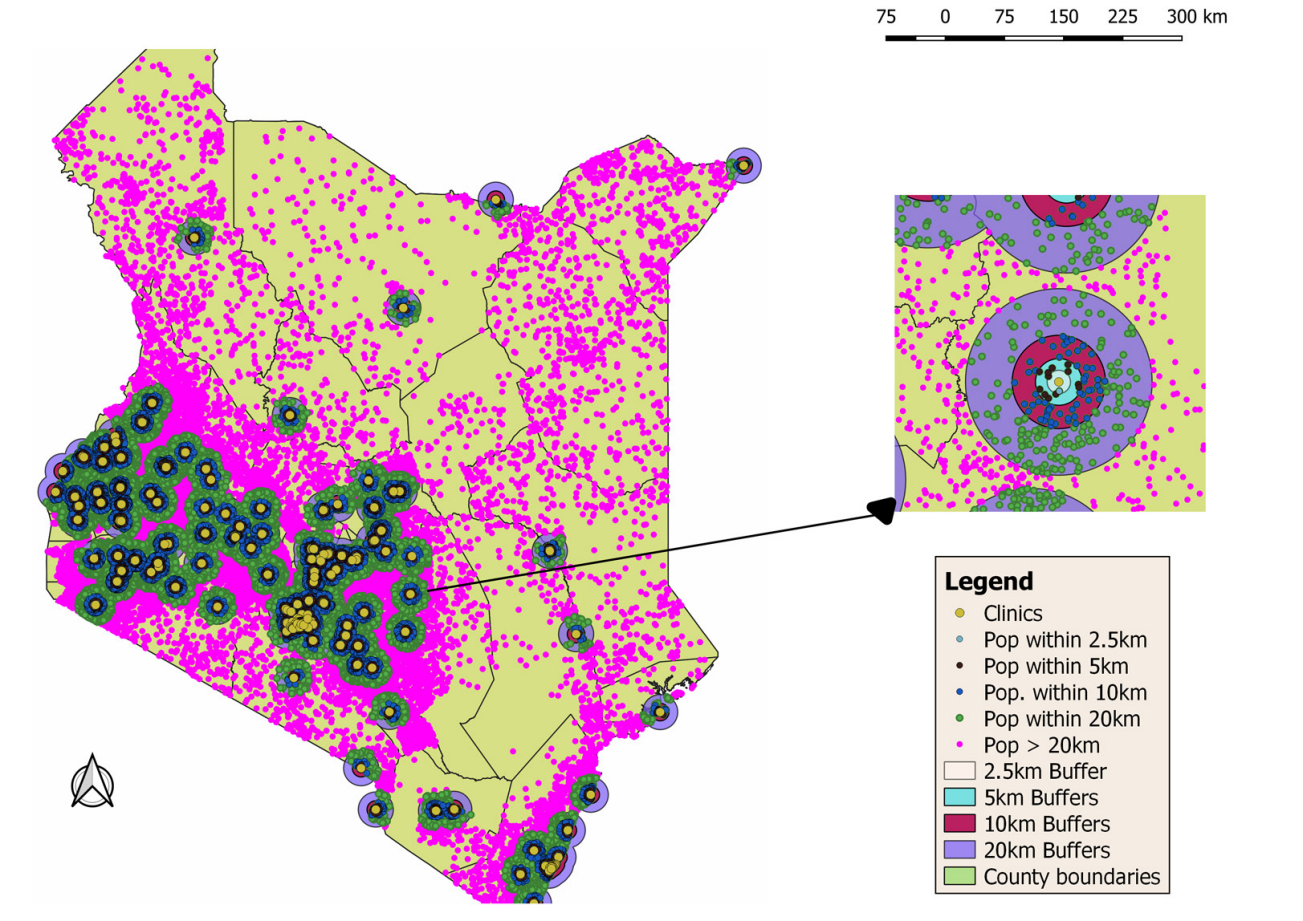
Population distribution by distance from the nearest dental clinic. (Map)

A distance-based analysis to determine how far people were from dental care was undertaken. At higher magnification these population numbers at the specified distances from dental services could be visualized (Figure 4, inset).

Counties situated in the middle third of the country had a high concentration of people living within shorter distances (2.5–5 km) of a dental facility, whereas those in the northern and north-eastern regions recorded a higher population living within the greater distances (10-20 km or greater). For instance, approximately half of those within 2.5 km were in Nairobi County alone, and an overall 73% at the same distance were in six of the more urbanized counties including Mombasa and Kisumu. In general, only 58% (22.4 million) of the Kenyan population were within 20 km radius or less from the nearest dental facility. Of these, 10% (4.1 million) were within 2.5 km, 8% (3.2 million) within 5 km radius, 17% (6.4 million) within 10 km, and 23% (8.7 million) within 20 km. The distance to public and private clinics correlated to population size (Supporting Figure 5).

**Figure 5:**
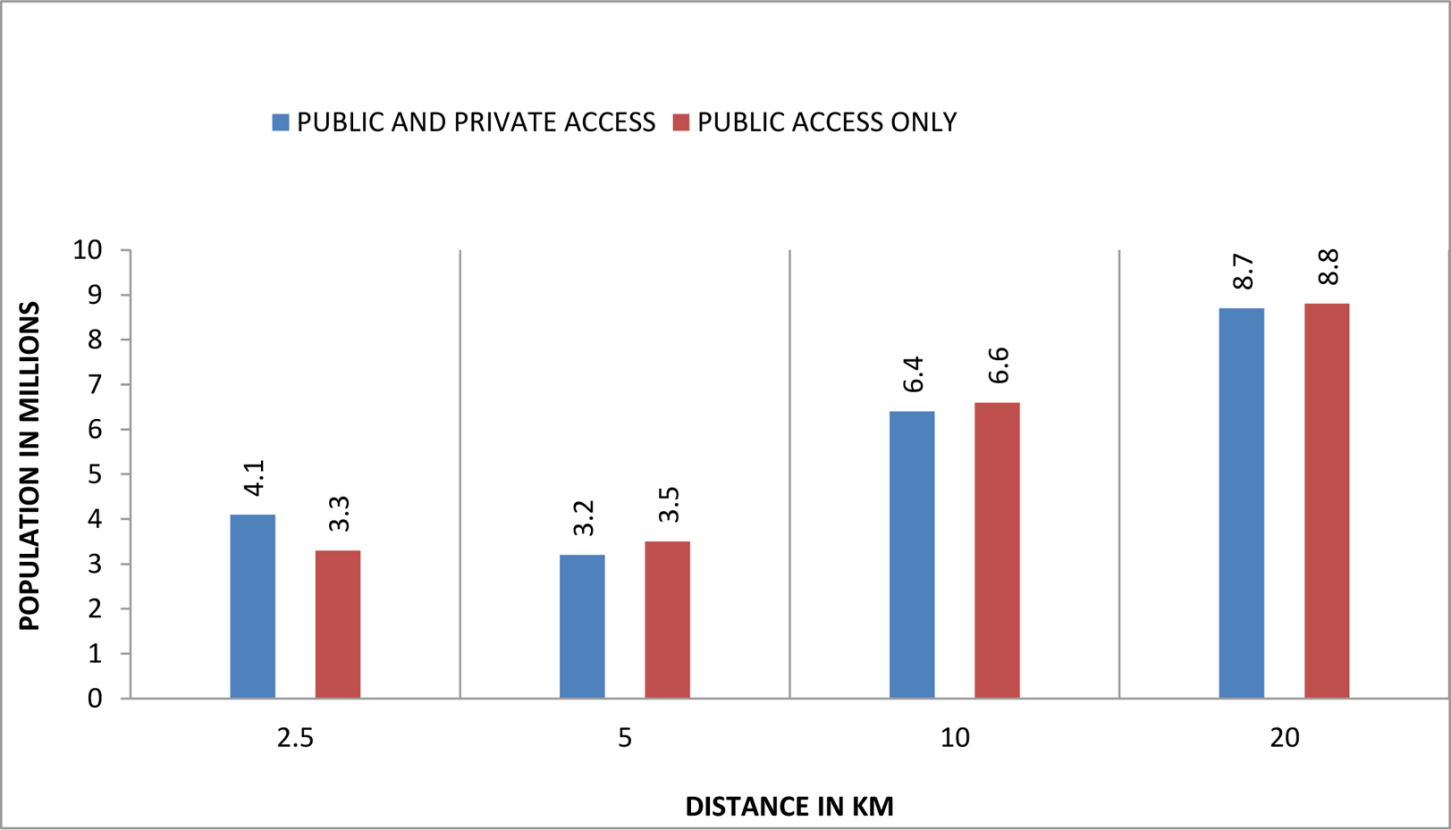
Population distribution by distance from the nearest dental clinic. (Graph)

## Discussion

This is the first study to explore and visualize the distribution of the OHW and dental care facilities in Kenya across the 47 counties using GIS technology. Our findings highlight not only major disparities in the distribution of the dental workforce and facilities across the country, they also illustrate these differences based on population densities/size, urbanization levels, and geographical accessibility.

This study has shown that the distribution of dentists and allied professionals per county was, to a large extent, directly proportional to the urbanization and population levels. That there were less public dental facilities compared to private was not surprising due to better financial rewards and career satisfaction associated with private practice in the highly populated urban areas, unlike the rural areas. Although government-sponsored dental facilities were found in both urban and rural areas, they were more in urban areas. Nairobi County, one of the two most urbanized and among the wealthiest according to the 2009 population census [22], hosted approximately 40% of all dental professionals and also had the bulk of both public and private dental facilities. It also recorded the highest dentist to population ratio of approximately 1:9,000, becoming the only county having close to the WHO minimum recommended ratio of 1:7,000. Mombasa, the only other county with 100% urbanization level and the second most densely populated, accounted for only 5% of the OHW. Coming in third was Uasin Gishu County, which had 8%. Although not among the highly urbanized or populated counties, the relatively high numbers of oral health professionals and dental facilities in Uasin Gishu County can be attributed to the presence of the Moi Teaching and Referral Hospital (MTRH), the second largest referral hospital in Kenya, and the Moi University School of Dentistry, the only other dental school in the country. Narok, on the other hand recorded the lowest dentist to population ratio despite being more populated and urbanized compared to some of the other counties such as Marsabit. One plausible explanation is its proximity to more urbanized counties such as Nakuru, Kiambu and Nairobi, where a larger proportion of its population can still access health care through cross travel. This scenario highlights one of the major weaknesses of utilizing only predefined units in area-based measures; they may not reflect cross-area travel between units, resulting in underestimation [23].

Although inequities in oral health care access appears to be more pronounced in LMICs than in HICs, all countries experience greater shortages in rural/economically disadvantaged areas. For instance, several states of the United States and provinces of Canada have reported disparities between metropolitan and rural counties [24,25,26,27,28], and a study in Australia reported up to 90% of dentists being in urban zones with dental specialists acutely lacking in rural areas [29]. In Africa, a Nigerian study reported that rural dentists comprised 20% of all the dentists in the year 2000 [30]. The strong correlation between number of dental professionals and level of urbanization has largely been attributed to the high population densities coupled with high per capita income in urban areas [26,31]. This is particularly true for economies where oral health is largely a private enterprise and services are rendered on a fee-for-service basis. As aforementioned, large proportions of rural dwellers are often faced with serious financial difficulties to afford these out-of-pocket payments, and therefore are not considered as being a conducive market for dental practices. Unfortunately, it has been shown that people in these areas have the worst oral health problems and therefore are in greater need of these services than are the affluent in large cities [32]. To mitigate this ‘inverse care-law’, where those with greater need of health care are least likely to receive it, training more doctors with a rural background would be beneficial as the probability of them returning to work in the rural areas is approximately twice as great as their urban-raised peers [33,34,35]. Suggestions have also been made to place students, with or without a rural background, in rural hospitals as part of their training to increase their retention in rural areas [32].

This study also compared the accessibility of dental care by distance across the 47 counties and further highlights the existing disparities in distribution of dental service provision. Once again, most of the Kenyan population living in the more urbanized and highly populated counties had better geographical access to dental care. Not surprisingly, Nairobi County, with over 63% of all private practices and the bulk of public dental facilities accounted for half of all Kenyans living within 2.5 km of dental services. In the majority of the largely rural counties, where people rely on only one or two public facilities, very few had access within shorter distances. In general, only 11% of the Kenyan population was within a 2.5 km radius of a dental clinic, 19% within a 5 km and 35% within a 10 km radius, whereas greater than 40% of all Kenyans were not even within 20 km. This is in contrast to reports from two previous Kenyan studies by Noor et al., [36,37] which established that 60% and 82% of the sample populations in 2003 and 2004, respectively, were within 5 km of a public health medical facility – further evidence that general health care has been prioritized over oral health care. Additionally, dental service accessibility appears more advanced in HICs. For example, McGuire et al., [38] found that 50% and 70% of Australian patients living in outer metropolitan areas were within 6 km and 10 km, respectively, of a clinic, and although the focus of their study was on government emergency dental care, it was still a good reflection of the situation in overall dental care within the public sector, which half of the time is emergency related. Longer distances mean that populations in counties that are already economically disadvantaged and struggling to meet the high dental treatment costs must also worry about travel costs. As a result, they face an increased risk of unmet dental needs.

This study is not without its limitations. First, dental professionals’ data from both the KMPDC and the MOH had gaps and inaccuracies. We made significant efforts to mitigate this limitation by cross-checking data from previous registers, gazette notices, government reports and other online platforms. Where information could not be verified from other sources, the dental professional was not included in the analysis resulting in the available sample of dentists of 737 being reduced to only 658 (89%). Furthermore, the present study focused only on dental professionals practicing in Kenya as of 2013. Demographics and distribution data for future workforce planning will have to take cognizant of the ever continuous evolving dental workforce.

Measurement of access in the present study mainly employed area-based measures to analyze the differences in access within and between counties. The use of these large administrative units assumes that whole populations within each county have similar access to services when in fact variations are known to exist. In relation to distance, this study only focused on geographic access without factoring in other aspects that must also be considered in policy formulation. For instance, a population may be considered to have short distances to a dental facility but may face other barriers such as affordability and poor road infrastructure. Conversely, availability of reliable transportation may reduce or eliminate the effect of long distances.

Lastly, for some of the private clinics, information on the exact location, including the building and or street name was unavailable, while for some public facilities, especially in remote areas, the names and exact locations were not recognizable on Google maps. As a result, some of the geographical coordinates used for mapping were only an estimation with reference to the nearest recognized building or street, and in a few instances, mapping was not possible.

In conclusion, the use of GIS in healthcare research is dramatically increasing in the recent decades. However, in Africa, many countries are facing challenges in its adoption in their health care system. A majority are still lacking the necessary hard- and software and, even where it does exist, there is an acute paucity of high quality geocoded data/GIS datasets and digital data on health resources, population, utilization, treatments and outcomes for health service research [23]. They also lack proper leadership, resources and expertise to fully implement it within the health sector. Despite these set-backs, for the first time, our research team mapped the distribution of the OHW in Kenya and demonstrated the existing maldistribution of dental professionals and facilities that affects dental service access in most counties, especially those that are less urbanized and are sparsely populated. Our findings could be a good guide for health planners in these new government units. Both the national and county governments need to take advantage of the devolved health system to improve dental care access, through prioritization of oral health care in governance, enhanced public-private partnerships for service delivery, improved working environment to attract personnel in rural facilities, and development of strategies to strengthen health workforce information systems for efficient and continuous audit of the OHW. We recommend that future OHW studies take into account workforce productivity, population demographic trends, oral disease patterns, and the variations in oral health needs within and between counties for effective workforce policy development. The measure of access should include time and cost of travel to further demonstrate the existing disparities.

## Additional File

The additional file for this article can be found as follows:

10.5334/aogh.3903.s1 Kenyan Dental practices location data-2013. Global positioning data (longitudes and latitudes) of the public and private practice locations included in this study.
